# Design of an epitope‐based peptide vaccine against *Cryptococcus neoformans*

**DOI:** 10.1002/2211-5463.13858

**Published:** 2024-07-17

**Authors:** Ibtihal Omer, Isra Khalil, Ahmed Abdalmumin, Philisiwe Fortunate Molefe, Solima Sabeel, Islam Zainalabdin Abdalgadir Farh, Hanaa Abdalla Mohamed, Hajr Abdallha Elsharif, ALazza Abdalla Hassan Mohamed, Mawadda Abd‐Elraheem Awad‐Elkareem, Mohamed Salih

**Affiliations:** ^1^ Department of Therapeutic Drug Monitoring Laboratory National Center for Kidney Diseases and Surgery Khartoum Sudan; ^2^ Department of Microbiology, Faculty of Medical Laboratory Science Sudan University of Science and Technology Khartoum Sudan; ^3^ Biomedical Research Institute Sudan National University Khartoum Sudan; ^4^ Hair and Skin Research Laboratory, Department of Medicine, Division Dermatology, Groote Schuur Hospital University of Cape Town Cape Town South Africa; ^5^ Department of Pathology, Faculty of Health Sciences, Institute of Infectious Diseases and Molecular Medicine (IDM) University of Cape Town South Africa; ^6^ Faculty of Dentistry University of Khartoum Sudan; ^7^ General Administration of Quarantine and Animal Health Regional Training Institute Khartoum Sudan; ^8^ Department of Biotechnology, Faculty of Science and Technology Omdurmam Islamic University Sudan; ^9^ Department of Biotechnology Ahfad University for Women Omdurman Sudan; ^10^ Department of Biotechnology Africa City of Technology Khartoum Sudan

**Keywords:** *Cryptococcus neoformans*, epitope, glucuronoxylomannan, *in silico*, mannoprotein, vaccine

## Abstract

*Cryptococcus neoformans* is the highest‐ranked fungal pathogen in the Fungal Priority Pathogens List (FPPL) released by the World Health Organization (WHO). In this study, through *in silico* simulations, a multi‐epitope vaccine against *Cryptococcus neoformans* was developed using the mannoprotein antigen (MP88) as a vaccine candidate. Following the retrieval of the MP88 protein sequences, these were used to predict antigenic B‐cell and T‐cell epitopes via the bepipred tool and the artificial neural network, respectively. Conserved B‐cell epitopes AYSTPA, AYSTPAS, PASSNCK, and DSAYPP were identified as the most promising B‐cell epitopes. While YMAADQFCL, VSYEEWMNY, and FQQRYTGTF were identified as the best candidates for CD8+ T‐cell epitopes; and YARLLSLNA, ISYGTAMAV, and INQTSYARL were identified as the most promising CD4+ T‐cell epitopes. The vaccine construct was modeled along with adjuvant and peptide linkers and the expasy protparam tool was used to predict the physiochemical properties. According to this, the construct vaccine was predicted to be antigenic, nontoxic, nonallergenic, soluble, stable, hydrophilic, and thermostable. Furthermore, the three‐dimensional structure was also used in docking analyses with Toll‐like receptor (TLR4). Finally, the cDNA of vaccine was successfully cloned into the *E. coli* pET‐28a (+) expression vector. The results presented here could contribute towards the design of an effective vaccine against *Cryptococcus neoformans.*

## Graphical abstract

Mannoprotein 88 (MP88) has been identified as a virulence factor for *Cryptococcus neoformans* and it is involved in the induction of cell‐mediated immune responses. In this study, MP88 is targeted to predict B‐cell and T‐cell epitopes using various *in silico* approaches to construct a multi‐epitope vaccine design against *Cryptococcus neoformans*. Image created via https://www.stylar.ai.

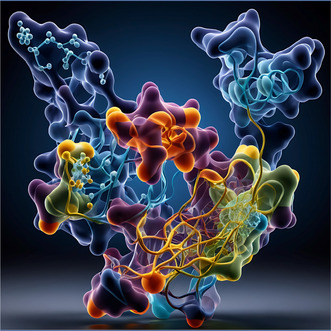

**Abbreviations**
AIDS: acquired immunodeficiency syndrome
Arg: arginine
Asp: aspartic acid
AUC: area under curve
CAI: codon adaptation index
CD: cluster of differentiation
cDNA: complementary deoxyribonucleic acid
CNS: central nervous system
CTLs: cytotoxic T cells
DC: dendritic cell
DTH: delayed‐type hypersensitivity
FPPL: fungal priority pathogens list
GC: guanine – cytosine
Glu: glutamic acid
GRAVY: grand average of hydropath*y*
GXM: glucuronoxylomannan
HLA: human leukocyte antigens
HTLs: helper T lymphocytes
IC50: half maximal inhibitory concentration
IEDB: immune epitope database and analysis resource
IFNγ: interferon gamma
IL: interleukins
kDa: kilodalton
Lys: lysine
MHC: major histocompatibility complex
MP: mannoproteins
NCBI: National Center for Biotechnology Information
OTR: organ transplant recipients
PTMs: post transcriptional modifications
TLR: toll‐like receptor
TNFα: tumor necrosis factor‐alpha
VLP: viral‐like particle
WHO: World Health Organization

Cryptococcosis is an infectious fungal disease caused by two pathogenic encapsulated *Cryptococcus* yeasts species, which may lead to fatalities in immunocompromised patients; these include *Cryptococcus neoformans* and *Cryptococcus gattii* [1, 2]. *Cryptococcus neoformans* is the most predominant etiological species, recognized widely as the common cause of the disease in the world, whereas *Cryptococcus gattii* infection is reported only in tropical and subtropical regions [3, 4, 5]. Cryptococcal infections are acquired through inhalation of aerosolized spores and yeast cells, primarily targeting the lungs and central nervous system (CNS), causing pulmonary cryptococcosis and cryptococcal meningoencephalitis respectively, in AIDS patients. However, infection may spread to other organs, which include the eyes, prostate, skin, and bone [6, 7].

Cryptococcosis morbidity and mortality rates continue to rise exponentially: ~220,000 cases of cryptococcal meningitis occur annually [8], with over 180,000 reported deaths each year, of which 75% occur in sub‐Saharan Africa [9] and rarely in Northern Europe [10]. Cryptococcal meningitis is among the most poorly funded diseases in the world, receiving about 0.2% of relevant research and development funding [11].

Additionally, cryptococcosis is considered high risk for patients with hematological malignancies, and those undergoing immunosuppressive or steroid therapies [12, 13, 14]. More so, in organ transplant recipients (OTR) this disease exhibits a mortality rate between 20% and 40% [15, 16, 17].

Fungal infections are typically treated with antifungal drugs, such as amphotericin B and fluconazole. Although effective, several adverse effects of Amphotericin B, including nephrotoxicity, anemia, and electrolyte abnormalities have been reported. Hence, monitoring renal function and complete blood counts is necessary during administration [18]. Although 60–80% of fluconazole is excreted through the kidneys and 11% is eliminated via hepatic metabolism [19]; up to 10% of patients treated with the drug developed asymptomatic liver injury. [20]. In addition to the mentioned toxicity, drug resistance has been reported as the major challenge associated with fungal therapy [21, 22]. Therefore, there is a need for alternative pharmaceutical solutions, and the development of a vaccine against *Cryptococcus neoformans* has become inevitable.

Vaccines are the most reliable approach to protecting against certain diseases and preventing serious illnesses. There are several types of vaccines, including attenuated, inactivated, and viral‐like particle (VLP) vaccines [23]; these traditional vaccines require pathogen cultures, assays, and approaches that are laborious and costly [24]. Conversely, advances in computational biology and bioinformatics have enabled the rapid design of useful vaccines, employing the computational reverse vaccinology approach to identify antigenic surface proteins from genomic data [25]. It involves constructing multiple fragments (epitopes) from microorganism proteins to elicit both cellular and humoral immune responses while reducing the adverse effects [26].

*Cryptococcus neoformans* possesses several virulence factors, which include polysaccharide capsules around yeast cells, production of melanin and mannitol, and phospholipase B and protease [27, 28, 29, 30]. The capsule, its main virulence factor, is primarily composed of glucuronoxylomannan (GXM), with galactoxylomannans and mannoproteins (MPs) as minor components [31].

The polysaccharide components reportedly inhibit phagocytosis, suppress immune response by inhibiting chemokine and cytokine production, while mannoproteins are highly mannosylated, immunopotent antigens involved in the induction of cell‐mediated immune responses [32, 33, 34, 35, 36], delayed‐type hypersensitivity (DTH) [37, 38, 39], tumor necrosis factor alpha (TNFα) expression [40, 41], enhancement of IL‐12, IL‐6, IL‐10, IFNγ, IL‐8, and TNFα levels [42, 43, 44], and human dendritic cell (DC) activation [45, 46]. These findings suggest mannoproteins as important targets for vaccine design [32, 36, 47, 48]. Among all known mannoproteins, only MP98 and MP88 have been identified and cloned due to their ability to stimulate the production of T‐cell hybridomas [38, 49]. In light of this, we aimed to design a peptide vaccine by exploring *in silico* approaches for epitope prediction using mannoprotein MP88 as a vaccine candidate.

## Methods

**Fig. 1:**
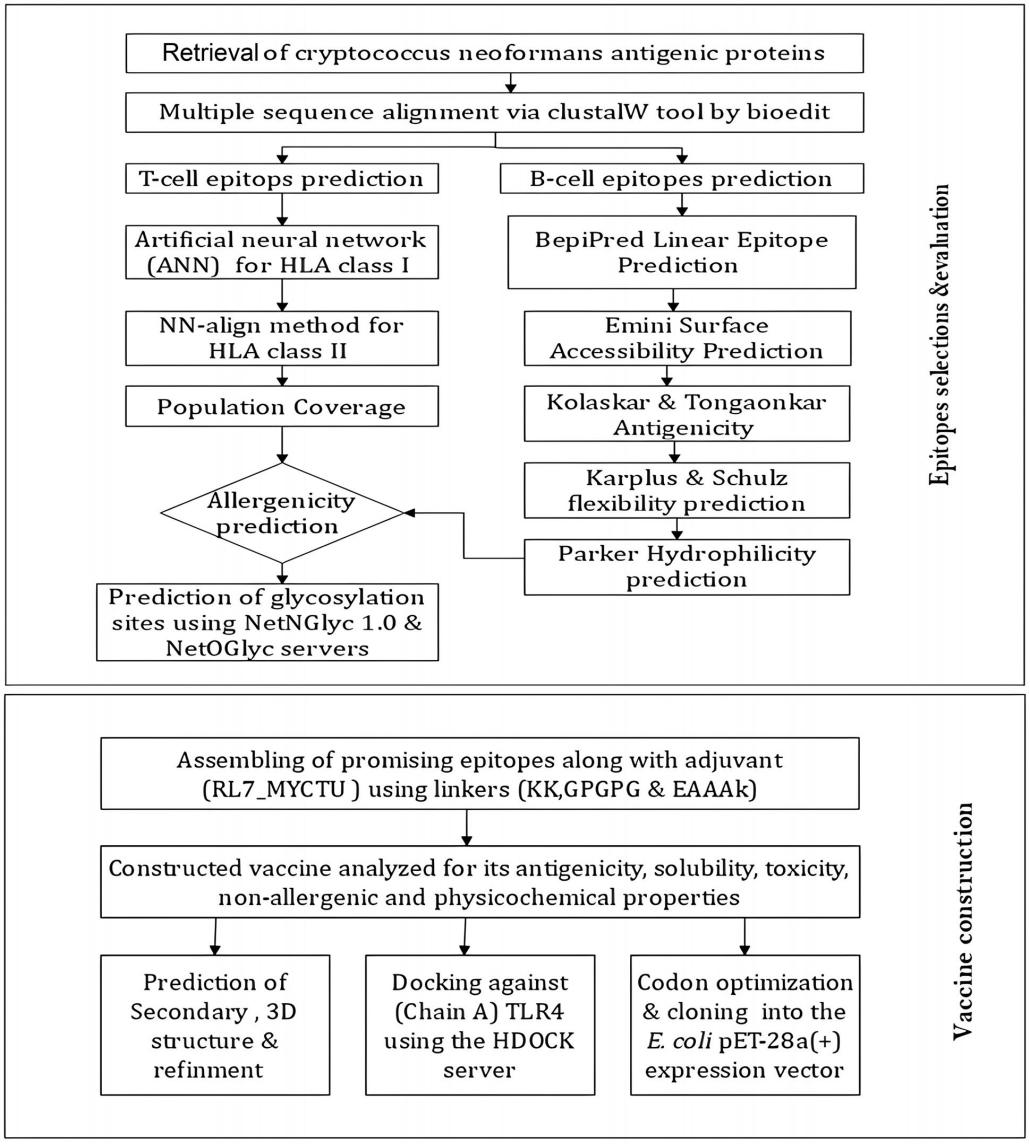
Flowchart for the different methodological steps involved in the multi‐epitope vaccine construct against cryptococcus neoformans, including essential tools and software. The method starts with the retrieval of protein sequence and multiple sequence alignment followed by epitope prediction and evaluation. construct of vaccine by assembling of epitopes along with adjuvant, evolution of vaccine then secondary and tertiary structure of vaccine prediction, refining of molecular docking. It ends with codon adaptation and in silico cloning.

A flow chart depicting the overall procedure for the development of a peptide vaccine for *Cryptococcus neoformans* is presented in Fig. 1.

### Retrieval of *Cryptococcus neoformans* antigenic proteins and alignments

A total of 39 sequences of MP88 proteins with lengths of 378 amino acids were retrieved from the National Center for Biotechnology Information (NCBI) database (https://www.ncbi.nlm.nih.gov/). All retrieved sequences, submitted in June 2017, belonged to the genome sequencing and analysis programs at the Broad Institute of MIT and Harvard. Sequences were downloaded in FASTA format and used for further analysis (Appendix S1). The molecular weight and amino acid composition of MP88 were determined using bioedit software v. 7.2.5 (Ibis Biosciences, Carlsbad, CA, USA). Subsequently, multiple sequence alignments via clustalw (EMBL, Heidelberg, Germany) were performed, and the conserved regions among the aligned MP88 protein sequences were identified using bioedit. Epitope conservation was determined using the Epitope Conservancy Analysis tool [50]. Tools for the analysis of epitope sets were obtained from the Immune Epitope Database and Analysis Resource (IEDB).

### Prediction of antigenic B‐cell epitopes

To predict the linear B‐cell epitopes, which is computationally more feasible than conformational epitopes, a full‐length sequence from accession number XP_012046953 was submitted to the bepipred Linear Epitope Prediction tool [51] at IEDB with a threshold value of 0.350. The bioedit sequence alignment editor was used to determine and select conserved epitopes, while the surface accessibility of each conserved epitope was investigated using the emini Surface Accessibility Prediction tool [threshold = 1.000] [52]. The Kolaskar and Tongaonkar Antigenicity tool was used to predict antigenicity [threshold = 1.016] [53], the Karplus and Schulz flexibility prediction tool (threshold = 1.013) was used to predict flexibility [54], and for hydrophilicity the Parker Hydrophilicity prediction tool (threshold = 2.418) [55] was used with varying window size from 4 residues to 30 residues for all tools. Thereafter, potential glycosylation sites on the selected regions were predicted, using netnglyc 1.0 (https://services.healthtech.dtu.dk/services/NetNGlyc‐1.0/) for N‐linked glycosylation sites using different thresholds (0.5, 0.32, 0.75, 0.90), where 86% of glycosylated and 61% of nonglycosylated residues were identified, with an overall accuracy of 76% [56]. Furthermore, NetOGlyc (https://services.healthtech.dtu.dk/services/NetOGlyc‐4.0/) for O‐linked glycosylation sites, only the sites with scores higher than 0.5 are predicted as glycosylated, of which 76% of glycosylated and 93% of nonglycosylated residues were predicted [57]. Only epitopes that were indirect contact with glycosylation sites were selected.

### T‐cell epitope prediction

HLA class I and class II T‐cell epitope predictions from conserved sequences were performed using IEDB tools, while the artificial neural network (ANN) method was used to predict 9‐mer HLA class I epitopes [58, 59]. The T‐cell HLA class II epitopes were identified using the NN‐align method [60]. Furthermore, all conserved epitopes that bind to alleles with scores of less than 500 for half‐maximal inhibitory concentrations (IC50) were selected for further analysis. The prediction of glycosylation sites was done by using the same methodology as mentioned in the previous step.

### Population coverage

The Population Coverage Analysis tool of IEDB was used to predict population coverage of the epitope across worldwide populations [61]. This tool is designed to calculate population coverage in different regions of epitopes based on the distribution of known MHC alleles to which epitopes bind. Predicted MHC‐I and MHC‐II epitopes of MP88 were analyzed using the Population Coverage Analysis tool in IEDB and the calculation option was set to class I and class II.

### Prediction of allergens

The allertop 2.0 (https://www.ddg‐pharmfac.net/AllerTOP/) server was used to predict allergenicity of selected epitopes. The server identifies allergens based on physicochemical properties of proteins, predicts route of exposure such as through food, inhalation, or toxins with high sensitivity. The sensitivity (94%) justifies its ability to identify new, structurally diverse allergens compared to those known [62].

### Vaccine construction

The most promising epitopes meeting the vaccine design criteria such as immunogenicity, surface accessibility, hydrophilicity, flexibility, conservancy, and nonallergenic were selected for final vaccine construction. The multi‐peptide vaccine candidate was constructed by linking the adjuvant to the epitopes. The RL7_MYCTU sequence (strain: ATCC 25618/H37Rv) used as an adjuvant was retrieved from the UniProtKB reviewed (Swiss‐Prot) Database (https://www.uniprot.org/uniprotkb/P9WHE3/entry). EAAAK, CPGPG, and KK are the types of linkers used to construct the vaccine sequence.

### Prediction of physiochemical, antigenic, allergenic, solubility, and toxic properties for vaccine constructs

the expasy protparam tool (Swiss Institute of Bioinformatics, Lausanne, Switzerland) was used to predict the physiochemical properties; it predicts the molecular weight, theoretical pI, instability, half‐life (*in vitro*), aliphatic index, and grand average of hydropathy values (GRAVY) of the input multi‐epitope vaccine sequence.

Antigenicity was assessed by the VaxiJen 2.0 server and the AllerTop 2.0 server was used to evaluate the allergenicity of vaccine constructs. Toxicity was predicted using the toxinpred 2.0 server (https://webs.iiitd.edu.in/raghava/toxinpred2/batch.html). Lastly, solubility was predicted using the Soluprot server (https://loschmidt.chemi.muni.cz/soluprot/).

### Structure prediction, refinement, and docking with the receptor of vaccine construct

The secondary structure prediction was carried out using the SOPMA server (https://npsa‐prabi.ibcp.fr/cgi‐bin/npsa_automat.pl?page=/NPSA/npsa_sopma.html). It uses the amino acid sequence to characterize secondary structures such as the alpha helix, beta sheets, beta turn, and random coils. Next, the tertiary structure was predicted by to 3Dpro in scratch protein predictor server (https://scratch.proteomics.ics.uci.edu/). The 3D structure of a vaccine construct was refined using the Galaxy Refine web server (https://galaxy.seoklab.org/cgi‐bin/submit.cgi?type=REFINE) and visualized by ucsf chimera v. 1.17.3. The HDOCK web server (http://hdock.phys.hust.edu.cn), which supports protein–protein and protein–DNA/RNA docking based on a hybrid algorithm of template‐based modeling and *ab initio* free docking was used to dock the vaccine construct with the human Toll‐Like Receptor4 (TLR4) (Chain A) (PDB ID:4G8A).

### Codon adaptation and *in silico* cloning

Codon adaptation and *in silico* cloning were performed to express the final vaccine construct in the *E. coli* (strain K12) host, since codon usage optimization demonstrated differences between human and the *E. coli* strain. The purpose of cloning was to ensure the expression of the vaccine construct in the selected host. The Java Codon Adaptation Tool (jcat) server (https://www.jcat.de/Start.jsp) was used for reverse translation of the vaccine construct protein sequence into the DNA sequence. The rho‐independent transcription termination, prokaryote ribosome binding site, and cleavage site of the restriction enzyme were avoided. In the JCAT, codon adaptation index (CAI) score is 1 and a score above 0.8 is considered as good. The favorable GC content of a sequence range is between 30% and 70% [63]. Thereafter, HindIII and EcoR1 restriction enzymes cleavage site sequences were introduced at the N‐terminal and C‐terminal regions of the DNA sequence generated by the server. Finally, the SnapGene restriction cloning module was used to insert the DNA sequence into pET28a (+) vector between the HindIII and EcoR1.

## Results

### Retrieval of *Cryptococcus neoformans* antigenic proteins and alignments

**Fig. 2:**
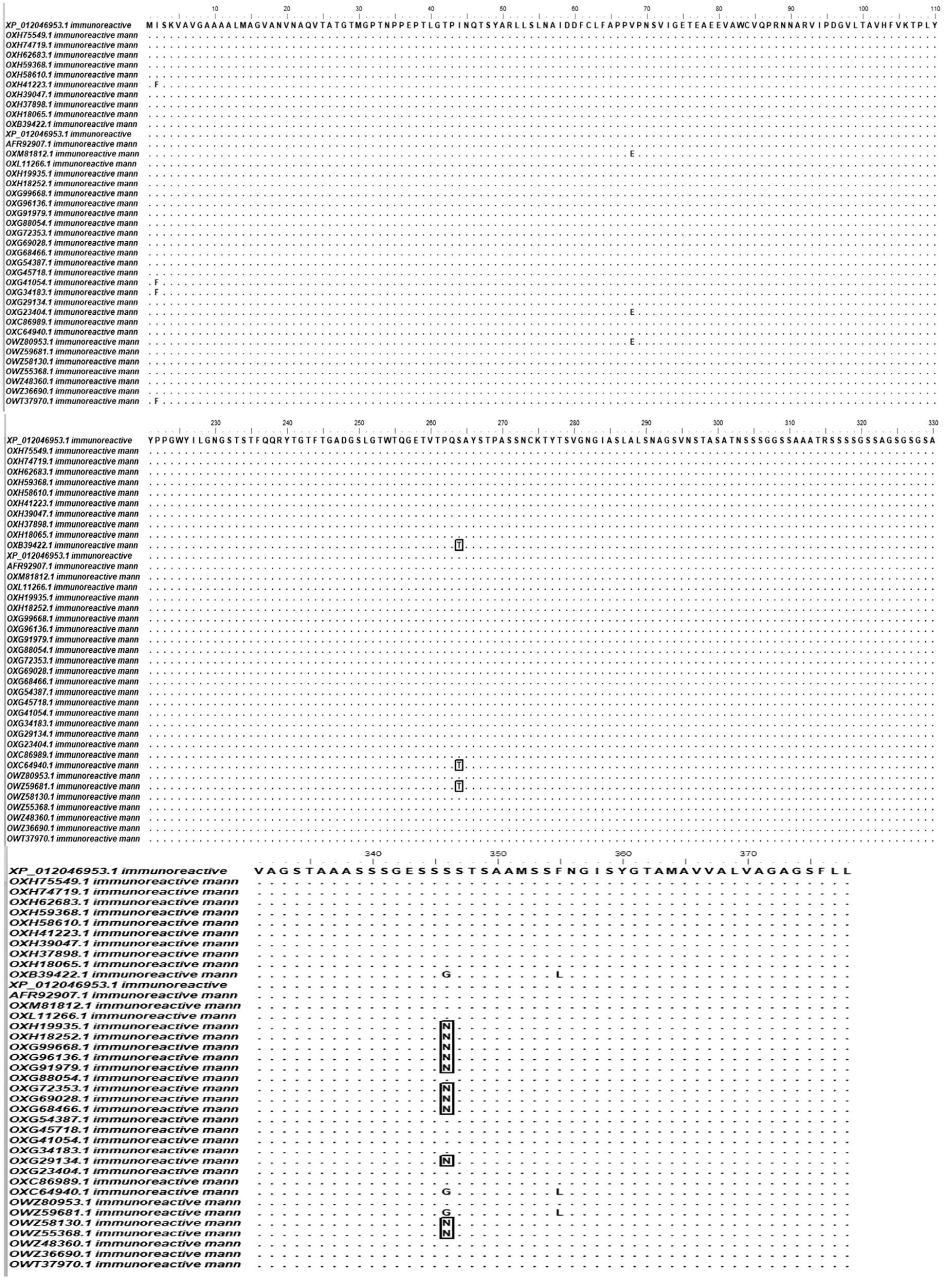
Multiple sequence alignments of MP88 using BIOEDIT software v. 7.2.5. via CLUSTALW. dots indicated the four conserved regions, located in position 3‐67 (length 65), position 69‐263 (length 195), position 265‐345 (length 81), and position 356‐378 (length 23) and letters within‐ and without rectangle indicated non‐conserved regions.

Multiple sequence alignments via clustalw showed four conserved regions, located in position 3–67 (length 65), position 69–263 (length 195), position 265–345 (length 81), and position 356–378 (length 23) among the aligned MP88 protein, sequences of the regions were then identified using bioedit, as shown in (Fig. 2).

### B‐cell epitope prediction

The *in silico* prediction of B‐cell epitopes using the bepipred tool led to the identification of 11 conserved peptides. A threshold of 0.571 was used as the cutoff point in the analysis; the results are shown in Table 1.

**Table 1:** Eleven predicted conserved antigenic B‐cell epitopesin MP88.

Start	End	Peptide/Conserved	Length
24	47	VTATGTMGPTNPPEPTLGTPINQT	24
69	79	PNSVIGETEAE	11
88	93	RNNARV	6
125	159	QSGDEGGELDPHGATGLGNPVGGNVTTNATGSDVS	35
182	187	STYSAA	6
206	223	DYTADSFTECDGDSAYPP	18
231	237	GSTSTFQ	7
241	263	TGTFTGADGSLGTWTQGETVTPQ	23
265	275	AYSTPASSNCK	11
293	345	GSVNSTASATNSSSGGSSAAATRSSSSGSSAGSGSGSAVAGSTAAASSSGESS	53
347	350	STSA	4

**Fig. 3:**
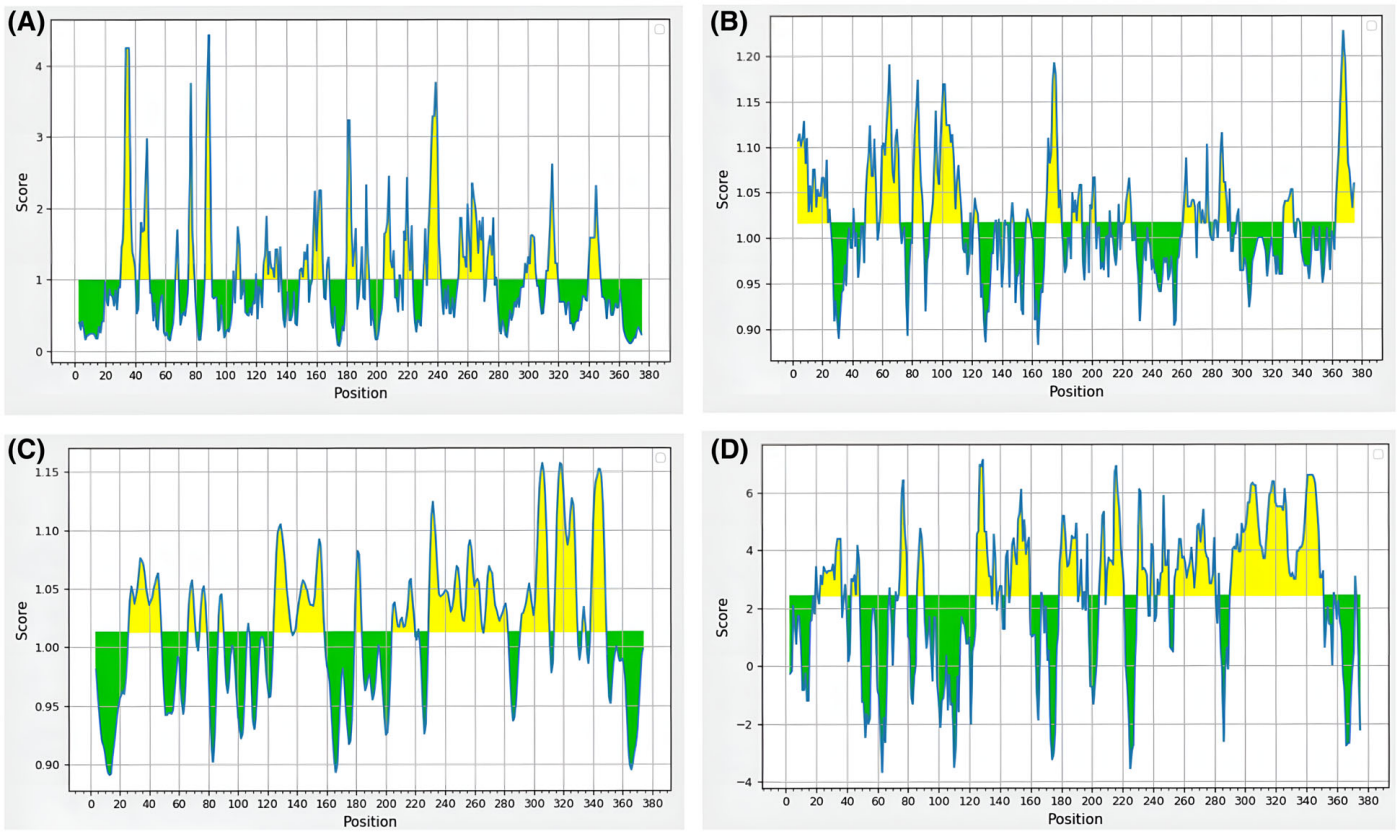
B‐cell epitope characteristics identified using different tools, including (A) surface accessibility, (B) antigenicity, (C) flexibility, and (D) hydrophilicity. The score of each residue is plotted on a Y‐axes and X‐axes correspond to the residue positions of MP88 protein sequence. The residues with scores higher than the threshold were predicted to be part of an epitope, as indicated in yellow color.

Emini surface accessibility predictions were employed to assess the surface accessibility of potential B‐cell epitopes. The results demonstrated that the average surface accessibility areas of the protein were 1.000, with both maximum and minimum accessibility of 4.429 and 0.065, respectively. All regions with predicted values equal to or greater than the default threshold of 1.000 are potentially at the surface. These results are shown in Table 2 and Fig. 3.

**Table 2:** The selected Linear B cell epitopes according to their surface accessibility, antigenicity, flexibility, and hydrophilicity result.

Start	End	Epitope	Length	Emini threshold = 1.000	Antigenicity threshold = 1.016	Flexibility threshold = 2.418	Hydrophilicity threshold = 1.013
90	93	NARV	4	1.038	1.024	0.594	2.4
182	185	STYS	4	1.784	1.023	0.615	4.075
183	186	TYSA	4	1.345	1.037	0.595	2.975
69	72	PNSV	4	1.087	1.059	0.636	2.975
265	268	AYST	4	1.345	1.036	0.64	2.975
266	269	YSTP	4	2.059	1.036	0.662	2.975
269	272	PASS	4	1.233	1.038	0.649	4.3
272	275	SNCK	4	1.015	1.032	0.649	5.15
218	221	DSAY	4	1.557	1.026	0.605	4.175
219	222	SAYP	4	1.441	1.075	0.629	2.2
220	223	AYPP	4	1.663	1.088	0.643	1.1
259	262	TVTP	4	1.05	1.066	0.649	2.2
260	263	VTPQ	4	1.26	1.093	0.682	2.4
37	40	EPTL	4	1.4	1.018	0.639	1.475
133	136	LDPH	4	1.273	1.071	0.645	1.25
295	298	VNST	4	1.014	1.02	0.669	3.75
338	341	ASSS	4	1.068	1.025	0.702	5.4
182	186	STYSA	5	1.451	1.032	0.793	3.68
183	187	TYSAA	5	1.094	1.042	0.773	2.8
265	269	AYSTP	5	1.674	1.042	0.844	2.8
266	270	YSTPA	5	1.674	1.042	0.857	2.8
218	222	DSAYP	5	1.937	1.033	0.827	3.76
219	223	SAYPP	5	1.794	1.073	0.827	2.18
271	275	SSNCK	5	1.095	1.028	0.843	5.42
258	262	ETVTP	5	1.464	1.023	0.844	3.32
259	263	TVTPQ	5	1.464	1.056	0.868	2.96
36	40	PEPTL	5	1.743	1.028	0.845	1.6
132	136	ELDPH	5	1.775	1.027	0.845	2.56
133	137	LDPHG	5	1.014	1.032	0.819	2.14
294	298	SVNST	5	1.094	1.018	0.866	4.3
182	187	STYSAA	6	1.185	1.037	0.911	3.417
**265**	**270**	* **AYSTPA**	**6**	**1.367**	**1.046**	**0.974**	**2.683**
266	271	YSTPAS	6	1.813	1.037	0.988	3.417
207	212	YTADSF	6	1.265	1.017	0.957	2.117
**218**	**223**	* **DSAYPP**	**6**	**2.421**	**1.038**	**0.941**	**3.483**
258	263	ETVTPQ	6	2.049	1.022	0.99	3.767
35	40	PPEPTL	6	2.178	1.034	0.985	1.683
**265**	**271**	* **AYSTPAS**	**7**	**1.483**	**1.041**	**1.039**	**3.229**
266	272	YSTPASS	7	1.967	1.033	1.061	3.857
**269**	**275**	* **PASSNCK**	**7**	**1.119**	**1.039**	**1.063**	**4.471**
265	272	AYSTPASS	8	1.632	1.037	Not available	3.638
268	275	TPASSNCK	8	1.327	1.022	Not available	4.563
266	274	YSTPASSNC	9	1.144	1.047	Not available	3.933
267	275	STPASSNCK	9	1.46	1.021	Not available	4.778
214	222	ECDGDSAYP	9	1.312	1.019	Not available	4.856
215	223	CDGDSAYPP	9	1.171	1.043	Not available	4.222
266	275	YSTPASSNCK	10	1.862	1.035	Not available	4.11
214	223	ECDGDSAYPP	10	1.651	1.023	Not available	4.58
35	44	PPEPTLGTPI	10	1.498	1.02	Not available	1.51
265	275	AYSTPASSNCK	11	1.524	1.038	Not available	3.927
212	223	FTECDGDSAYPP	12	1.363	1.019	Not available	3.483
211	223	SFTECDGDSAYPP	13	1.5	1.019	Not available	3.715

* Most promising B‐cell epitopes.

The Kolaskar and Tongaonkar [53] antigenicity prediction method functions on the basis of physiochemical properties of amino acids and their abundances in experimentally identified epitopes. The average antigenicity value was 1.016, with a maximum of 1.227 and a minimum of 0.883. All values equal to or greater than 1.016 indicated potential antigenic determinants. The results of predictions for all conserved predicted B‐cell epitopes are shown in Table 2 and Fig. 3.

**Fig. 4:**
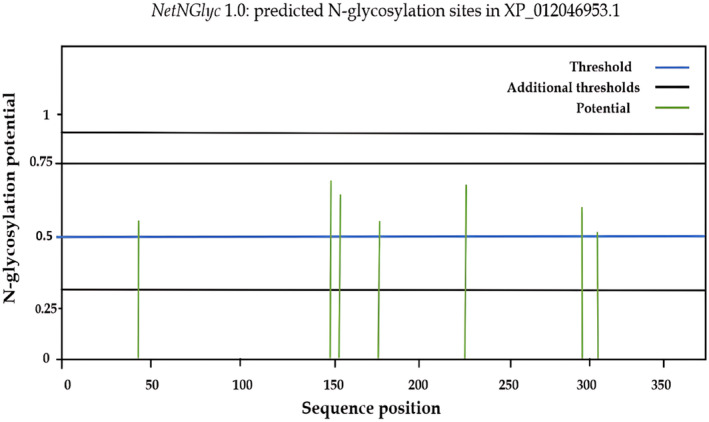
The graph illustrates predicted N‐glyc sites across the reference protein chain (*x*‐axis represents protein length from N‐ to C‐terminal). A position with a potential (vertical green lines) crossing the threshold (horizontal blue line at 0.5) is predicted glycosylated. Additional thresholds are shown at 0.32, 0.75, and 0.90 by horizontal black lines.

To investigate epitope flexibility, the Karplus and Schulz [54] flexibility prediction tool was used, with a default threshold value 1.013 (Table 2 and Fig. 3), Parker Hydrophilicity prediction scores were calculated based on peptide retention times during high‐performance liquid chromatography (HPLC). The average predicted hydrophilicity was 2.418, with a maximum score of 7.029 and a minimum value of 3.914. Any epitope hydrophilicity scores equal to or more than 2.418 signified a potential hydrophilic epitope. (Table 2 and Fig. 3). The most promising B‐cell epitopes DSAYPP, AYSTPA, PASSNCK, and AYSTPAS, which were not located in the glycosylation sites, are illustrated in (Fig. 4).

### T‐cell epitope prediction

The artificial neural network (ANN) method was used to predict T‐cell epitopes in the MP88 reference protein. The three most promising conserved epitopes, YMAADQFCL, VSYEEWMNY, and FQQRYTGTF, were selected based on their IC50 values of less than 50 nM and 500 nM, which ensures the selection of MHC‐I molecules for which the selected epitopes show higher and intermediate affinities. Among these three epitopes, the 9‐mer epitope YMAADQFCL interacts with nine MHC‐I alleles, and of these nine alleles, HLA‐A*02:01 is predicted to most highly interact with an epitope (IC50 = 5.34). These conserved epitopes are likely to ensure the more successful immunizations than other conserved epitopes (Table 3). The allergenicity of these three epitopes was evaluated using allertop 2.0. VSYEEWMNY and FQQRYTGTF are nonallergenic while YMAADQFCL is allergenic.

**Table 3:** The selected potential CD8 T cell epitopes along with their interacting MHC class I alleles with affinity IC50 <50 and 500 by the ANN method. And epitope antigenicity and conservancy result.

Start	End	Epitope	HLA class I alleles	IC50^a^	Epitope conservancy %	Antigenicity	Antigenicity score
158	166	VSYEEWMNY	HLA‐A*03:01 HLA‐A*11:01 HLA‐A*29:02 HLA‐A*30:02 HLA‐B*15:01 HLA‐C*12:03	253.17 359.89 95.3 65.45^b^ 165.34 365.45	100	Antigen	1.5789
166	144	YMAADQFCL	HLA‐A*02:01 HLA‐A*02:06 HLA‐A*29:02 HLA‐B*15:01 HLA‐B*39:01 HLA‐C*03:03 HLA‐C*07:02 HLA‐C*12:03 HLA‐C*14:02	5.34^b^ 16.99 400.69 438.65 39.46 9.18 382.21 262.28 78.34	100	Antigen	1.8119
236	244	FQQRYTGTF	HLA‐A*02:06 HLA‐A*24:02 HLA‐B*15:01 HLA‐B*15:02 HLA‐C*07:02 HLA‐C*12:03 HLA‐C*14:02	255.61 418.17 19.09^b^ 41.68 306.14 101.78 166.51	100	Antigen	1.0969

^a^ ANN IC50 is the inhibitory concentration needed for successful binding of peptide to MHC molecule by the Artificial Neural Network method. The lower number of IC50 is the better

^b^ Epitopes reveal higher binding affinities.

We used the NN‐align tool for MHC class II peptide binding predictions. YARLLSLNA, ISYGTAMAV, and INQTSYARL showed the highest binding affinities of the conserved epitopes and had IC50 < 50. In addition, INQTSYARL is predicted to interact with 14 MHC‐II alleles, while ISYGTAMAV and YARLLSLNA are both forecasted to interact with 12 MHC‐II alleles (Table 4). Finally, ISYGTAMAV and INQTSYARL are predicted to be nonallergenic, while YARLLSLNA was a predicted allergen, and all of them are not involved in glycosylation sites (Fig. 4).

**Table 4:** The three potential CD4^+^ T‐cell epitopes along with their interacting MHC class‐II alleles with affinity IC50 <50 by the NN‐align method and epitopes conservancy result.

Start	End	Core	Peptide	IC50	HLA class II alleles	Epitope conservancy %
354	368	ISYGTAMAV	SFNGISYGTAMAVVA	5.5	HLA‐DPA1*01:03/DPB1*02:01, HLA‐DQA1*01:02/DQB1*06:02, HLA‐DQA1*04:01/DQB1*04:02, HLA‐DQA1*05:01/DQB1*03:01, HLA‐DRB1*01:01, HLA‐DRB1*03:01, HLA‐DRB1*04:01, HLA‐DRB1*04:04, HLA‐DRB1*07:01, HLA‐DRB1*09:01, HLA‐DRB1*13:02, HLA‐DRB1*15:01	100
355	369		FNGISYGTAMAVVAL	5.3^a^		
353	367		SSFNGISYGTAMAVV	7.2		
356	370		NGISYGTAMAVVALV	6.8		
39	53	INQTSYARL	TLGTPINQTSYARLL	9^a^	HLA‐DPA1*01/DPB1*04:01, HLA‐DPA1*01:03/DPB1*02:01, HLA‐DPA1*02:01/DPB1*01:01, HLA‐DPA1*02:01/DPB1*05:01, HLA‐DRB1*01:01, HLA‐DRB1*04:05, HLA‐DRB1*07:01, HLA‐DRB1*08:02, HLA‐DRB1*09:01, HLA‐DRB1*11:01, HLA DRB1*13:02, HLADRB1*15:01, HLA‐DRB4*01:01, HLA‐DRB5*01:01	100
41	55		GTPINQTSYARLLSL	10.2		
40	54		LGTPINQTSYARLLS	11.6		
38	52		PTLGTPINQTSYARL	12.5		
44	58	YARLLSLNA	INQTSYARLLSLNAI	4.9^a^	HLA DPA1*03:01/DPB1*04:02, HLADQA1*01:02/DQB1*06:02, HLA‐DRB1*01:01, HLA‐DRB1*04:01, HLA DRB1*04:04, HLA‐DRB1*04:05, HLA‐DRB1*07:01, HLA‐DRB1*08:02, HLA‐DRB1*09:01, HLA‐DRB1*11:01, HLA‐DRB1*15:01, HLA‐DRB5*01:01	100
47	61		TSYARLLSLNAIDDF	5.8		
43	57		PINQTSYARLLSLNA	5.9		
48	62		SYARLLSLNAIDDFC	7.2		

^a^ Highly binding affinity.

### Analysis of population coverage

Population coverage predictions were performed with the maximum possible population size. For MHC class I, YMAADQFCL, VSYEEWMNY, and FQQRYTGTF showed high levels of predicted presence in the world population based on the IEDB population coverage tool. The population coverage of YMAADQFCL was the highest at 69.75%, while VSYEEWMN and FQQRYTGTF displayed lower percentages of 58.78% and 54.11%, respectively. The average population coverage overall was 93.01% (Table 5).

**Table 5:** Population coverage calculation results for MHC‐I.

Population/Area	Class I		
	Coverage^a^	Average_hit^b^	PC90^c^
World	93.01%	2.43	1.11
Average	93.01	2.43	1.11
Standard deviation	0.0	0.0	0.0

^a^ Projected population coverage

^b^ Average number of epitope hits/HLA combinations recognized by the population

^c^ Minimum number of epitope hits/HLA combinations recognized by 90% of the population.

For MHC‐II, the ISYGTAMAV epitope displayed the highest population coverage of 74.39%, while YARLLSLNA and INQTSYARL showed lower coverage amounts of 69.46% and 63.94%, respectively. The average population coverage was 81.94% (Table 6). Ten alleles are not available in the IEDB, and therefore were not included in these calculations (Appendix S2).

**Table 6:** Population coverage calculation results for MHC‐II.

Population/Area	Class II		
	Coverage^a^	Average_hit^b^	PC90^c^
World	81.94%	25.59	3.32
Average	81.94	25.59	3.32
Standard deviation	0.0	0.0	0.0

^a^ Projected population coverage

^b^ Average number of epitope hits/HLA combinations recognized by the population

^c^ Minimum number of epitope hits/HLA combinations recognized by 90% of the population.

### Vaccine construction

**Fig. 5:**
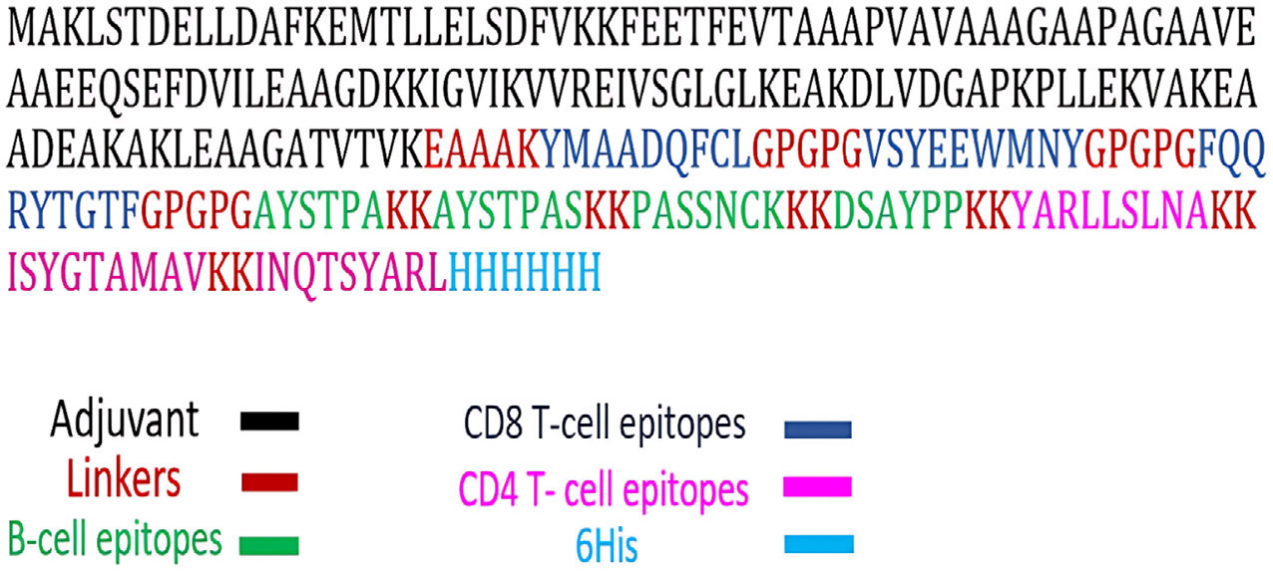
Representation of construct vaccine including linkers, adjuvants, B‐cell epitopes, CTL and HTL epitopes. EAAAK linkers connect the adjuvants, GPGPG linkers connect CTL epitopes, KK linker connects HTL epitope and B‐cell epitopes, Histidine‐Tag to final construct.

The sequence was made up of the CTLs, HTLs, and B‐cell epitopes predicted above to construct a multi‐epitope vaccine sequence. The KK linker was used to link B‐cell and HTL epitopes, whereas the GPGPG linkers were used for CTL epitopes. The RL7_MYCTU sequence used as an adjuvant to increase the immunogenicity of the vaccine and linked to the first CTL epitopes by EAAAK linker, and the C‐terminal joined with HHHHHH (6HIS) linker, which indicated stability for vaccine structure and the ease of purification (Fig. 5).

### Prediction of physiochemical, antigenic, allergenic, solubility, and toxic properties for vaccine constructs

the protparam tool was used to calculate the physicochemical properties of vaccine, and the results showed that the final construct vaccine was composed of 248 amino acid residues, with a molecular weight of 26 kDa and a theoretical isoelectric point value (pI) of 8.59. The total number of negatively (Asp + Glu) and positively (Arg + Lys) charged residues was 30 and 33, respectively. The assessed half‐life was 30 h (mammalian reticulocytes, *in vitro*), >20 h (yeast, *in vivo*) and >10 h (*E. coli*, *in vivo*). The instability index obtained was 23.57, while the aliphatic index was 74.19, and the grand average of hydropathicity (GRAVY) was −0.295. Findings from the Vaxigen 2.0 server, demonstrated that the designed vaccine construct was antigenic, with an overall prediction score of 0.8660 (threshold = 0.5). On the other hand, allertop 2.0 and toxinpred 2.0 tools classified the construct as nonallergenic and nontoxic to humans, respectively, while the Soluprot server classified the construct as soluble with a prediction score of 0.731 (threshold = 0.5).

### Structure prediction, refinement, and docking with the receptor of vaccine construct

**Fig. 6:**
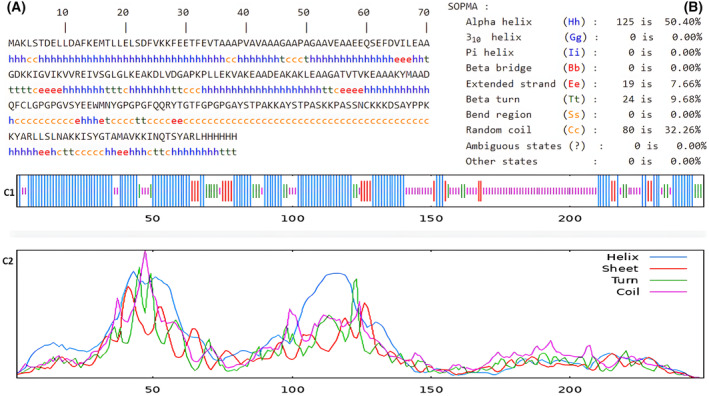
Prediction of secondary structure of multi‐epitope vaccine construct by SOPMA server. **(A)** The sequence of secondary structure of vaccine with length 248 aa, the Alpha helix (h), Extended strand (e), Beta‐turn (t), Random coil (c). **(B)** The percentage of each secondary vaccine element. **(C1)** A graphical represent the target sequence with exact position of secondary elements, i.e., alpha helices (blue color), extended strand (red color), beta turn (green color) and random coli (purple color). **(C2)** The score curve of alpha helices, extended strand, beta turn and random coli in each predicted stat.

**Fig. 7:**
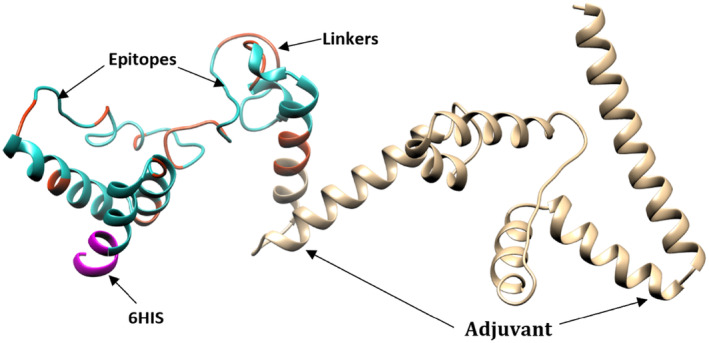
The 3D structure of designed vaccine generated by 3Dpro in scratch protein predictor server and visualized by Chimera 1.17.3.; tan color represents adjuvant, 6HIS represents by magenta color, vaccine epitopes represented by light sees green color, and sienna color representing linkers.

The results on the SOPMA server demonstrated that the secondary structure contains 248 amino acids, 50.4% alpha helix, 7.66% extended strands, 9.68% beta sheets, and 32.26% random coils, as depicted in Fig. 6. The 3D model of the vaccine construct, predicted by the 3Dpro server, is shown in Fig. 7. The vaccine construct was refined using the galaxy refine tool to improve the physical quality of the structure; this resulted in nine models of the vaccine, and according to the Parameter Rama favored score of 97.6; and visualization by the chimera tool, model 9, was the most refined.

**Fig. 8:**
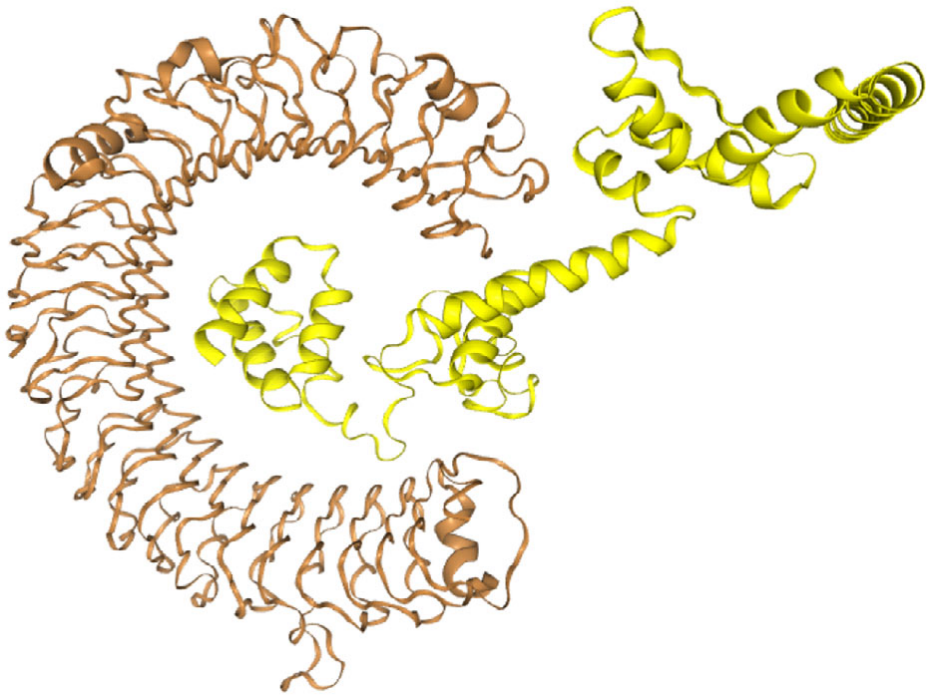
Visualization of docking HDOCK results for constructed vaccine‐TLR4 complex. The constructed vaccine structure is shown as the cartoon and sticks in yellow. TLR4 (Chain A) structure is shown as the cartoon and sticks in copper color.

The docking analysis of the vaccine construct against Toll‐like receptor 4 (chain A) using the HDOCK server showed a strong binding energy of −301.40 kcal·mol^−1^. The energy scores gained for the receptor were minimal among all the other predicted docked complexes showing the highest binding affinity. A low (negative) energy showed a stable system (Fig. 8).

### Codon optimization and *in silico* cloning of vaccine candidate

**Fig. 9:**
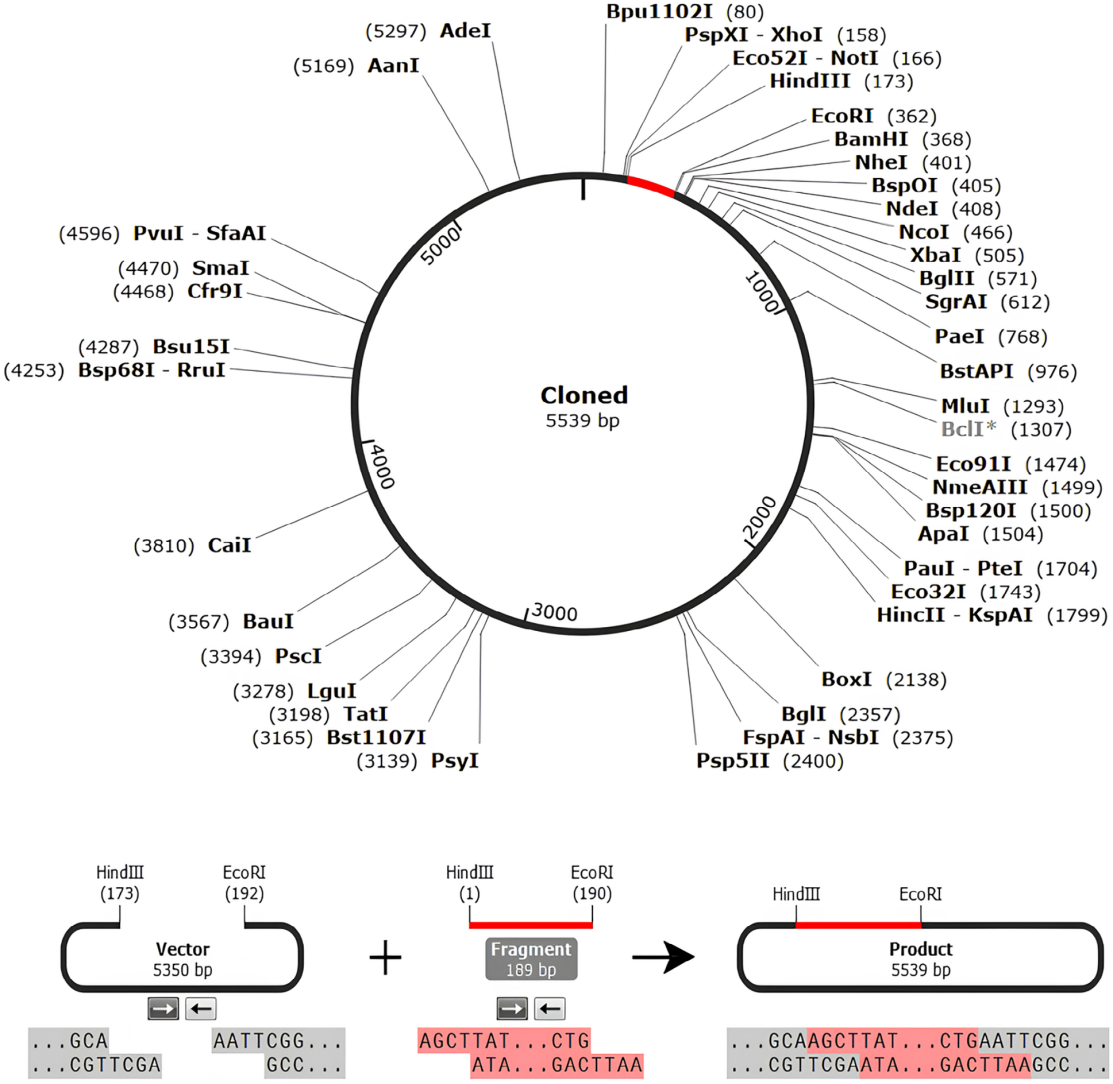
In silico restriction cloning of the multi‐epitope vaccine sequence into the pET‐28a (+) expression vector using SnapGene software, the red part represents the vaccine's gene coding, was cloned between the HindIII and EcoR1 sites, and the black circle represents the expression vector.

The DNA sequence of the construct vaccine as calculated by the JCAT server was found to be 744 bp in length. The predicted Codon Optimization Index (CAI) value was 0.9, while the GC content of the improved sequence was 50%. The restriction enzymes HindIII and EcoR1, tagged onto the N and C terminal of the insertion fragment (vaccine construct), was then cloned into the pET28a (+) vector plasmid. The precloning construct was 5350 bp, whereas after insertion, the vector size increased to 5539 base pairs (Fig. 9) and was illustrated using SnapGene.

## Discussion

In this study we successfully designed multi‐epitope vaccines for *Cryptococcus neoformans* targeting its immunogenic mannoprotein MP88 by using immunoinformatic tools. The predicted peptide vaccine is potentially characterized by easy production, the stimulation of effective immune responses, and no potential for infection [64].

B‐cell epitopes are regions in pathogenic antigens recognized by B‐cell receptors or antibodies to induce specific humoral responses [65]. Linear B‐cell epitopes of MP88 protein were identified using the bepipred linear epitope prediction tool (IEDB). The fully conserved epitopes were tested using different immunoinformatic tools to predict surface accessibility, antigenicity flexibility, and hydrophilicity. Based on the high scores in these tests, the epitopes AYSTPA, AYSTPAS, PASSNCK, and DSAYPP appeared to be the most promising B‐cell epitopes.

The accuracy of IEDB B‐cell epitope prediction tools consists of area under curve (AUC) values ranging from 0.6 to 0.7, which are better when compared to other existing online methods; using these tools, class I AUC values greater than 0.9 and class II values greater than 0.87 represented a perfect prediction [66].

CD8^+^ T‐cells recognize antigens of a pathogen only when combined with MHC‐I molecules, leading to cytotoxic responses [67]. The ANN method precisely determined three promising T‐cell epitopes within MP88 in relation to their (IC50) values, epitopes with IC50 values <50 nM were considered to have high, <500 nM intermediate, and <5000 nM low affinity [68]. The epitope VSYEEWMNY showed intermediate binding affinity with HLA‐A*30:02, while YMAADQFCL and FQQRYTGTF revealed high binding affinity with HLA‐A*02:01 and HLA‐B*15:01, respectively. These epitopes interact with the most predominant HLA class I alleles in humans, including HLA‐A*02:01 [69, 70]. Hence, these epitopes may potentially provide protection against cryptococcal infections for 93% of the world's population if used as a vaccine.

CD4^+^ T‐cells orchestrate adaptive immune responses in humans [71]. Specific interactions with high binding affinity of epitopes and HLA allele class II molecules unleash protective and specific adaptive immune responses. Our identified T‐cell CD4^+^ epitopes were YARLLSLNA, ISYGTAMAV, and INQTSYARL revealed high binding affinity to different HLA class II alleles with IC50 <50 nM. Immunization with these three epitopes could potentially protect ~81% of the world's population.

One of the fundamental approaches to enhance vaccine immunogenicity is posttranscriptional modifications (PTMs) of vaccine candidates of protein, peptide, etc. Glycosylation is one of several types of PTMs [72]. There are two main types of glycosylation, N‐ and O‐glycosylation, in which saccharides are attached either to the amide group of an asparagine residue or to hydroxy amino acids, which are mainly serine and threonine residues. Glycosylation plays an important role in regulating T‐cell activity and highly glycosylated areas in proteins will downregulate the activity of these cells [73]; as a consequence, all selected epitopes in this constructed vaccine are not predicted to be in these glycosylation sites and this improves the chance of the host reacting to the native antigen after receiving this multi‐epitope vaccine.

The constructed vaccine performed by assembly promising B‐cell and T‐cell epitopes linked together using appropriate linkers along with adjuvants. GPGPG and KK linkers were included between the anticipated epitopes in earlier experiments to generate a vaccine with the best antigenicity possible [74] and EAAAK linker is adjusted to attach the adjuvant (RL7_MYCTU) with the first‐predicted CTL epitopes. These linkers play important roles in flexibility, assisting folding, and making the recombinant multi‐epitope vaccine structure more stable [75].

The physiochemical features of constructed vaccines revealed that the vaccine's molecular weight is 26.27306 kDa, which is within the acceptable range for a subunit vaccine [76], and the isoelectric point value of 8.59 indicating that the vaccine is basic in nature. The score of instability index was 23.57, which was less than 40, and this classifies the vaccine as a stable protein. The aliphatic index was 74.19, which was >70 and this is indicates that a protein is thermostable over a wide temperature range. The GRAVY value for vaccine is calculated as the sum of hydropathy values of all of the amino acids divided by the number of residues in the sequence [77]. The average of hydropathicity (GRAVY) was −0.295, which classifies the vaccine construct as hydrophilic. This construct vaccine was also predicted to be antigenic, nonallergenic, nontoxic, and a soluble vaccine. The half‐life of the vaccine *in vitro* was calculated to be 30 h in mammalian reticulocytes, suggesting that it can remain viable for an adequate span of time to generate a potent immune response [78].

The SOPMA server was used to predict and analyze the constructed vaccine secondary structure, and the most common elements of the secondary structure in proteins are the alpha helix, where it represents 50.4%, and plays a major role in mediating protein–protein interactions [79]; beta sheets were revealed to be 9.68%; 7.66% extended strands, and 32.26% random coils, Following this, a homology modeling approach was utilized to construct the tertiary structure of the final designed vaccine and the galaxy refine tool was used to perform the refinement of the 3D protein structure ,where the ideal vaccine model was selected according to the Rama favored score of 97.6.

The vaccine was docked with the TLR‐4 receptor (chain A) to determine the vaccine's ability to elicit a significant immunological response. Toll‐like receptors (TLRs) are a family of cell surface receptors that enable phagocytic inflammatory responses to a variety of microbial products [80]. TLR4 is one of the main TLRs that are involved in sensing fungal components, such as O‐linked mannans, zymosan, phospholipomannan, and fungal DNA [81].

The expression of the vaccine construct in *E. coli* K12 analyzed by the *in silico* cloning and the jcat online tool where the codon optimization of the construct vaccine was reverse‐translated to its cDNA to ensure a successful expression in *E. coli* pET‐28a (+) expression vector. The GC and CAI values predicted were 50% and 0.9, respectively, resulting in a possibility of good expression of the vaccine construct in the *E. coli* K12 strain [74].

*Cryptococcosis* is the most prevalent fungal infection in immunocompromised individuals, especially in people living with HIV, cancer, or individuals who have recently undergone solid organ transplantations. Many deleterious complications of *Cryptococcus* infections, including severe pulmonary disease and fatal meningoencephalitis, have been reported [5, 82, 83, 84]. The high cost of treatment and clinical challenges, such as toxicity and antifungal resistance [85, 86], has led to the adoption of strong and prolonged preventive approaches. To date, there have been no approved vaccines for *Cryptococcosis*.

Previously generated killed vaccines against *Cryptococcus neoformans* have been unsuccessful, while live attenuated vaccines induce protective immunity in immunocompetent animals, but are not practical for immunocompromised hosts [87].

GXM, which is known as a poorly immunogenic antigen, is the sole component of the cryptococcal capsule. Multiple studies have shown that immunization with GXM‐based vaccines, even when GXM is conjugated to a protein or tetanus toxoid (GXM‐TT), fails to induce specific protective immune responses and often has deleterious effects [82, 88].

Multiple proposed vaccines are still undergoing experimental trials. A vaccine based on heat‐killed chitosan from the cryptococcal cell wall triggers the generation of robust protective immunity against virulent strains of *Cryptococcus neoformans* in mice, thus making this vaccine a candidate for use in humans [84]. Another experimental vaccine based on alkaline extracts from mutant cryptococcal strains packaged in glucan particles (GP) appears to provide significant protection in mice. However, identification of the active molecule is still in progress [88]. In addition, a GXM mimotope‐based vaccine may be promising [85, 88]. Indeed, focusing on the response triggered by a single epitope may enhance the risk of generating resistant variants [85].

These vaccine experiments are likely costly and do not consider the genetic diversity of *Cryptococcus neoformans* antigens and the potential world population coverage of a proposed vaccine, which have all been considered in this study.

Many reports have identified mannoproteins, the minor component of the *Cryptococcus neoformans* capsule, as strong potential vaccine candidates [38, 85] that may trigger protective Th1‐type immune responses against *Cryptococcus neoformans* infections. Notably, vaccination of mouse models with MPs and an R adjuvant produce an apparent T‐cell immune response, but not an antibody response [89].

This is the first example of an *in silico* approach for designing *Cryptococcus neoformans* epitope vaccines based on immunoinformatics tools and will help facilitate the manufacture of a specific, protective vaccine for immunocompromised patients around the world. A successful peptide vaccine should be composed of B‐cell and T‐cell epitopes. Peptide vaccines are poorly immunogenic when used alone [90]. Therefore, this constructed vaccine with adjuvants are suitable for improving *in vivo* T‐cell and antibody immune responses.

Despite the significance of the results from our studies, we have noted some minor issues. There is a need to expand the number of cryptococcal MP88 protein (378 aa) sequences analyzed to those from all parts of the world from global protein databases to reduce the margin of error. Calculations of some HLA class II alleles and epitopes for population coverage were missed; the immune simulation was not included in this research; and we considered it as a limitation that impacted the generalization of the results.

## Conclusion

B‐cell and T‐cell epitopes were predicted using different *in silico* tools; only epitopes that predicted conserved, antigenic, flexible, nonallergenic, and not located at glycosylation sites were used for the vaccine construction. Promising epitopes revealing high scores were joined to adjuvants using suitable linkers in the design of a multi‐epitope vaccine. Various tests were conducted to evaluate the construct vaccine and it was predicted to be antigenic, soluble, nontoxic, and nonallergenic, in addition to evaluation of the physiochemical properties.

The vaccine construct was docked against Toll‐like receptor 4 using the HDOCK server with an attractive binding energy of −301.40 kcal·mol^−1^. Finally, this construct vaccine was successfully expressed in the *E. coli* pET‐28a (+) vector. However, this vaccine needs to be further validated for its efficacy by *in vitro* and *in vivo* studies.

## Conflict of interest

The authors declare no conflicts of interest.

### Peer review

The peer review history for this article is available at https://www.webofscience.com/api/gateway/wos/peer‐review/10.1002/2211‐5463.13858.

## Author contributions

IO wrote the introduction, method and discussion. IK wrote method and discussion. IO, IK, AA, SS, PFM, IZAF, HAM, HAE, AAHM, MAA, MS participated in the practical of method section. IO, AA, PFM, SS revised and approved the article. PFM carried out the proofreading and editing.

## Supporting information


**Appendix S1.** 39 sequences of MP88 (378 aa) retrieved from NCBI including reference sequence.

**Appendix S2.** Alleles are not available in the IEDB.

## Data Availability

Data used to support the findings of this study are available from the corresponding author upon request.
