# Supplementary material for: Design of an epitope‐based peptide vaccine against Cryptococcus neoformans
Source: FEBS Open Bio. 2024 Jul 17;14(9):1471–89. doi: 10.1002/2211-5463.13858 (PMC11492362; doi:10.1002/2211-5463.13858)
Supplement: Supplementary file 2 — Appendix S2. Alleles are not available in the IEDB. [file FEB4-14-1471-s001.docx]

- **Appendix S2:** Alleles are not available in the IEDB**.**

1. HLA-DQA1*05:01/DQB1*03:01
2. HLA-DQA1*01:02/DQB1*06:02
3. HLA- DPA1*01:03/DPB1*02:01
4. HLA-DPA1*01/DPB1*04:01
5. HLA- DPA1*02:01/DPB1*05:01
6. HLA-DRB4*01:01
7. HLA-DRB5*01:01
8. HLA-DQA1*04:01/DQB1*04:02
9. HLA-DPA1*03:01/DPB1*04:02
10. HLA-DPA1*02:01/DPB1*01:01
